# On the Anatomy and Diseases of the Urinary and Sexual Organs; Containing the Anatomy of the Bladder, and of the Urethra, and the Treatment of the Obstructions to Which These Passages Are Liable

**Published:** 1845-08

**Authors:** 


*On the Anatomy and Diseases of the Urinary and Sexual Organs; containing the anatomy of the Bladder and of the Urethra, and the treatment of the obstructions to which these passages are liable.* By G. J. Guthrie, F. R. S., Surgeon to the Westminster Hospital, and to the Royal Westminster Ophthalmic Hospital, &c. &c. From the third London edition. Philadelphia: Lee & Blanchard. 1845.

It was a quaint remark of the late Dr. Parrish, that "the cream of Surgery was contained within the pelvis," and certainly there are no diseases within the range of the science, which require for their successful management a more intimate acquaintance with the anatomy of the parts concerned, and a higher exercise of skill in operative procedures, than those which affect the urinary organs. There are, indeed, few complaints attended with an equal amount of risk and suffering, and in which the prompt interference of the Surgeon is more urgently demanded, and his efforts, if successful, more highly estimated, or more amply rewarded.

It is, therefore, with no little satisfaction, that we notice the work of Mr. Guthrie in an American dress, and at a cost which renders it accessible to all classes of physicians.

It is needless to bestow praise upon a book, from a writer who deservedly takes rank amongst the first of English Surgeons, and whose opportunities of observation and experience upon the subjects of which he treats have been so ample; we can only exhort our readers to possess themselves of it, and study its contents, and we can promise them an addition to their stock of knowledge, upon a difficult and perplexing subject.

The first two chapters are chiefly occupied with an exposition of the anatomy and relative position of the urinary organs, and with directions for using the catheter. On some anatomical points Mr. Guthrie's views differ from those generally entertained by Surgeons.

The parts about the neck of the bladder are thus described :

"The opening, or meatus, through which the urine passes, is the commencement of the neck of the bladder; it is situated at the lower portion of the anterior part, and opens internally almost abruptly, or with a little depression, but in no way resembling the funnel-like process to be observed in animals. If the bladder be cut across transversely, this opening will be seen resembling a perpendicular slit, although it is sometimes of a rounder form. Beneath it there is a space of a triangular form, evidently whiter and of a more condensed and elastic structure than any other part; the apex of this triangular space, *trigone* of the French, is formed by a slight projection at the very opening of the bladder, called its uvula, or *luette*, whilst the base of the triangle is marked by a strong whitish-colored band, which passes transversely across from side to side. The ureters open within the two extremities of this band, and from these two another more prominent line or band descends, inclining inwards, so as to meet like the letter Y, the point being inserted into and behind the uvula. The width of the base is about an inch and a quarter from the opening of one ureter to that of the other; and there is about the same distance from the apex of the triangle to the base. The ureters, descending from the kidneys, enter the back part of the bladder obliquely, pass between the longitudinal and spiral layers of fibres, and then proceed obliquely through the spiral or internal layer, to open near the extremities of the base of the triangle above described. The orifices of the ureters are surrounded by a peculiarly condensed and elastic substance which lies beneath the mucous lining of the bladder, and between it and the internal muscular coat. This superadded structure begins at the base of the triangle, inclines inwards as it advances towards the neck of the bladder, forms in a great measure the orifice, appears to be continuous in the passage forwards, and to constitute the elastic membrane of the urethra. The triangular space of the bladder being elastic, yields to a certain extent to any moderately dilating force, from which point it returns to its original state, on the removal of the extending power."

Mr. Guthrie's peculiar views in regard to the mechanism by which the reflux of urine through the ureters is prevented, during a state of distension in the organ, are thus expressed—

"The linear bands, descending from the ureter on each side, are composed of a substance partly muscular, partly elastic.

They have been called the muscles of the ureters, and are described as inserted, fleshy and tendinous, into the prostate gland. They appear to be inserted fibrous into and behind the uvula, but the fibres of the bladder generally vary much with regard to the manner in which they pass over or are inserted into the prostate; and I have no doubt that some fibres may be found passing into the posterior edge of the gland, as has been stated by Sir C. Bell. It has been hitherto presumed that the ureters have no valves at their orifices to prevent the reflux of urine into them after it has passed into the bladder, such an apparatus being unnecessary in consequence of the oblique manner in which they enter between the muscular layers of bladders, which compress and close them when it is distended, so as to prevent a reflux from taking place. It appears to me that this mechanism is intended for the very reverse object. The ureter opens on a peculiarly condensed structure, in order that the orifice may be constantly patulous; and the obliquity of its passage through and between the muscular coats of the bladder, is for the purpose of giving facility to its being pressed upon and closed when the viscus is in a distended state, in order to delay if not to prevent the further flow of urine into it from the kidney. When the bladder is contracted and empty, the urine passes readily into and gradually dilates it, until the desire for expulsion comes on and leads to its evacuation. A little more or a little less seems to have no influence in preventing the urine from finding its way in, the weight of the column descending from the kidney readily overbalancing to a certain point the resisting power of the coats of the bladder. When the bladder is distended, it no longer yields easily, the ureter is pressed upon by the muscular wall in its passage through it, and the further entrance of urine is in a great measure prevented. If the obstruction be long continued, the ureter above this part is gradually dilated from the size of a crow quill to that of a man's thumb, and even larger; the pelvis of the kidney increases in size, a low or chronic inflammation is induced, the secretory organs are pressed upon and partially removed, so that the kidney may become at last an almost empty bag separated by partitions, indicating only the former existence of its lobes. A total suppression of the secretion may, under such circumstances, take place at any time. The most remarkable example of the kind which has come under my observation, occurred in the case of a lady who suffered from a cancer of the uterus; the disease after a time extended towards the ureters, which at last were embraced and pressed upon by it as they entered the bladder. The lady, as this took place, began to suffer more from derangement in her urinary apparatus; the bladder was found ultimately, on passing the catheter, to contain little or no water; she fell into a state of low fever, became paralytic, afterwards comatose, and died. On examination, the orifices of the ureters were found in a sound state, although the ureters were impervious at the part where they were grasped by the diseased structure; above this they were greatly enlarged, and the kidneys were much diseased and sacculated. The peculiar manner in which the ureters enter into the bladder is, then, an admirable provision of nature for the purpose of preventing too great a distention of the bladder rather than of the ureters, for nature can accommodate herself for several days to a complete suppression of the secretion of urine, and for a very long time to a partial secretion of it. The natural quantity usually secreted varies from two pints to two and a half in the twenty-four hours, and when an obstruction takes place preventing its evacuation, the bladder may become considerably distended; but the same quantity will not be secreted during the second twenty-four hours as in the first, and there will be still less during the third, before which time relief ought to be given by operative means, if it should not occur otherwise. This provision of nature is therefore intended, I apprehend, to protect as far as possible the bladder and urethra, rather than the constitution of the patient; the bladder and urethra being more susceptible of mischief in a shorter time than the system at large. I am disposed to believe that the two bands on the triangular space, called the muscles of the ureters, are better fitted for keeping the part fixed, and for strengthening and for raising it up when necessary, than for keeping open, and in a straight line, the channel of the ureters."

Mr. Guthrie lays great stress upon the existence of an elastic structure at the neck of the bladder, and believes that many affections attributed to the enlargement of the prostate gland, arise from a defect in the elastic property of this part. As a consequence of this defect, stricture of the orifice of the neck of the bladder occurs, and at a later period, if not relieved, irregular muscular action is induced, the transverse and longitudinal muscular fibres of the bladder act with greater vigor, and during this augmented action the mucous coat sometimes becomes distended by the urine, and protrudes externally between the muscular fibres, thus producing vesical pouches.

The effect of these vesical pouches in forming a lodgement of stone, and upon the evacuation of the bladder in cases of retention of urine, is thus noticed.

"The commencement of an extra vesical pouch is thus formed, which goes on increasing, if the same cause continues which gave rise to it, until it attains considerable magnitude : the opening between the muscular fibres by which it began, is usually of a small size, leading to a large cavity, into which a stone may pass, and be fortunately shut up so as to give rise to no further inconvenience, and to the belief that the stone has been dissolved or passed. In other instances, the opening may be so large and in such a situation as to admit of the stone being struck by the point of the sound, although it will not be readily discovered or extracted after the operation for its removal has been accomplished. In all cases a quantity of urine may and will be received in these pouches, and various secretions may be poured into and retained in them. In some cases of this kind, after drawing off the urine by the catheter, and as I supposed emptying the bladder, I found I could still get more by passing the instrument in a certain direction, and in all probability into one of the pouches which had been thus formed. This is however an accidental circumstance, not commonly met with. If the bladder be emptied by the catheter in the erect position, and the patient be made to change it by lying down, retaining the catheter in its place, an additional quantity may run from the instrument, showing that one or other of these pouches has been emptied. A gentleman consulted me on account of a difficulty he had in passing his water, for which he used an elastic catheter twice a-day and sometimes thrice, with great relief. The symptom he complained of as most disagreeable, was, that after emptying his bladder in the erect position before going to bed, he soon after felt the desire to make water return, and on straining forcibly he could pass a small quantity. I desired him to use the catheter a second time when he felt this uneasiness, which he did, and obtained about three ounces more water. This led to the belief that he had pouches in his bladder, which were only emptied by change of position. I wished him to ascertain what position emptied them ; but he could not do this in a satisfactory manner, for a reason which appeared after death, namely, that there were several pouches, which could not at all be emptied by the same position. He obtained, however, considerable relief by first drawing off his water in the erect position, and then by lying down with the catheter in the bladder, and by changing his position from either side to his face (for these pouches rarely form on the fore part) he removed a further quantity; after which he obtained rest, until the pouches and the bladder were refilled, and the desire to discharge his water again became considerable. In another gentleman, the existence of one or more pouches of this kind became evident on injecting the bladder; twelve ounces of warm water could be thrown into it before much uneasiness was produced; but on drawing it off, ten ounces only could be obtained, and rarely the whole twelve even by any change of position. In this case there were five pouches, of different sizes; and there was also a peculiar symptom, which I had then met with in three others, without being able to account for it, and which may have depended on the same cause in all."

Mr. Guthrie's description of the structure and course of the urethra is exceedingly minute and accurate; a thorough knowledge of this route, being of the utmost importance to the practicioner who undertakes the introduction of the catheter. He considers any amputation of the *length* of the canal of little consequence in practice, its points of attachment and curves being the chief guides in the introduction of instruments: the natural obstacles which are to be met with in the passage of the catheter, and the means of avoiding them are also carefully pointed out.

Several chapters of the work are occupied with the consideration of the several varieties of stricture.

In the treatment of spasmodic stricture, Mr. Guthrie considers that the ordinary practice of giving hot baths, fomentations, anodynes, &c., before resorting to the catheter, should be reversed, and that the catheter should be first tried, as the most speedy means of affording relief, and if this fail, that the other measures should be instituted preparatory to a second trial.

By this course, whether the catheter succeed or not in the first instance, the patient's mind is more reconciled to the use of the other remedies after a trial with the instrument.

We cannot follow our author, through his admirable account of the formation and symptoms of permanent stricture—but must be content with calling attention to several important practical points in their treatment.

The following remarks on the true method of dilating a stricture are well worthy consideration—

"A stricture cannot be cured by dilatation until such time as a passage has been obtained through it sufficient to admit a small bougie; when one of a size that will pass without inconvenience is to be introduced, and allowed to remain for any time not exceeding an hour; and if the bougie is a little conical, the stricture may not only be completely filled by it, but moderately dilated. If the stricture is very irritable, the soft bougie may be grasped and marked by it, and the same thing will occur if the bougie be too large and too strongly forced into it. If the bougie is rather too large at the point, it will not proceed on meeting with the stricture, although sometimes, by a gentle pressure for two or three minutes, the stricture will gradually yield, and allow it to go through; but there is a probability that more irritation will follow this mode of proceeding than if a small one were first introduced for a quarter of an hour, and a larger one then made to take its place, which it will almost always readily do. This is, in fact, the principle on which dilatation should be conducted, and it will always be accomplished more safely and easily for the patient if done in this manner; for it is indisputable that a larger bougie can always be introduced if it follows a small one, easier than without such a precursor. The bougie should never be used as a mere dilating instrument oftener than every two days, and when the urethra is irritable only every three and sometimes four days. Proceeding in this manner the stricture gradually yields, and a bougie, whether made of plaster or of silver, having a proper curvature, and as large as the orifice will admit, will at last proceed through the whole passage without meeting with any obstacle; and it ought to be repeated at longer intervals, until the disposition for contraction seems to be removed, when the cure will often be complete. When a stricture has been more permanent or of long duration, the patient should be taught the manner of passing it, so that he may use it once a week, then once a fortnight, and at last once a month or quarter. In this way I have made many most successful cures, even where the patient at the beginning of the treatment could scarcely pass his water; and there can be no doubt of its being the best method of proceeding when it proves successful, for the inner membrane of the urethra is restored as nearly as possible to its former and natural state."

In regard to the use of Caustics in permanent stricture, Mr. Guthrie differs from some of the highest surgical authorities.

He considers that the strong prejudice which exists particularly against the nitrate of silver, is founded upon the abuse of the article; and recommends its use under particular circumstances as a valuable aid to dilatation. The chief advantage to be derived from the application is in the relief of spasm and irritation, and not from its caustic properties in destroying the morbid structure.

The following method of application is recommended by Mr. Guthrie, and is certainly liable to fewer objections than the use of the solid stick formerly employed.

"I have for many years," says our author, "restricted my efforts with the lunar caustic to its admitted utility in removing that degree of irritation in a part which approaches to inflammation, and having long since found the great advantage to be derived from it in the form of ointment in chronic inflammation of the inside of the eyelids, I use it in almost a similar manner for the removal of irritable spots in the urethra. I have for this purpose a hollow elastic bougie, made round at the point, and of the same size throughout. Within an inch of the end, a round or oval hole should be made as in a catheter, and the part beyond the hole and up to the point should be filled up. Into the hole a quantity of the unguentum argenti nitratis* is to be introduced, and when the hole of the hollow bougie is opposite the irritable spot, the stillet made of whalebone is to be pushed down or home, having been previously so within an inch, when the ointment is forced out into the urethra, to the surface of which it is to be applied by turning the instrument half round, or by passing it backwards and forwards. The quantity used must depend on the judgment of the surgeon, and the age of the ointment, which is always milder in its effects, from the decomposition which takes place as it becomes older. It gives no pain at the time of application, although it sometimes causes a heat and a slight sensation, to be shortly afterwards followed, in most instances, by great relief. It does not however answer in all cases, and is not recommended as a universal remedy for them, although I have never seen it do mischief. It is very efficient when properly applied in some of the worst of those cases which Lallemand has described, but which are so rarely seen in England, as affecting the posterior part of the canal, accompanied by unknown or almost imperceptible emissions of semen, which may be found in the urine. It is equally, indeed more useful in chronic irritation of the anterior part of the urethra which remains after a gonorhœa, when it may be applied on the surface of a soft bougie, and will often effect a cure when all other means fail."

Mr. Guthrie does not consider the effects of the potassa fusa as salutary, either as an allayer of irritation, or as a remedy for the destruction of hard and gristly parts, as the nitrate of silver, whilst it is open to nearly all the objections urged against the latter article; judiciously applied, however, he thinks it well adapted to some cases. In connection with the application of caustics, we find some interesting observations on the occurrence of

* The unguentum argenti nitratis is made of ten grains of finely powdered argentum nitratum, rubbed carefully up with one drachm of ung. cetacii and fifteen minims of the liquor plumbi diacetatis.

hemorrhage from the urethra, and the means of arresting it, which we cannot avoid transferring to our pages, knowing the difficulty which often occurs in the treatment of this accident:

"Hemorrhages from the urethra, after the application of caustic, are caused by the sloughs separating and leaving the cells of the corpus spongiosum exposed, or by the ulcerative process extending to some small vessels, the canals of which are partially opened. These, it is said, cease of themselves, although not until a great loss of life has been frequently sustained, and it has been recommended to let the parts alone. I apprehend, however, that they should be met and treated like hemorrhages from the same place from other causes.

The most alarming hemorrhages I have met with occurred from common causes, and were arrested by pressure on the perineum. A gentleman living in Cockspur-street, had had a catheter passed by a surgeon of great reputation and ability in the morning, without either pain or inconvenience. On his return home he found there was a considerable oozing of blood, an accident which may readily happen without any undue force having been applied, which continued during the day, and induced him to send in the evening for his surgeon, who was unluckily out of town; the bleeding increased in the night, and in the morning early I saw him. There were several tubs of ice and water in the room, all apparently containing a considerable quantity of blood; his face was deadly pale, the pulse scarcely perceptible; and he said he had bled a pailful, which was of course an exaggeration. The bleeding was arrested in a few minutes by pressure, and did not return.

A tradesman had passed a common soft bougie for himself, the point of which had caught on some small opening, and, it is presumed, had penetrated into it; he bled for two days and two nights, when I was desired to see him in Paddington-street. I found him kneeling in bed, and straining violently to pass his water, but which came with great difficulty, as the bladder contained a good deal of coagulated blood, which had passed backwards into it. He was as white as a sheet, and fell back in his bed, nearly insensible, almost as soon as I entered the room; having, as he said afterwards, passed several quarts of what (as it all coagulated) he considered to be pure blood; but as urine and blood coagulate together when out of the body in equal proportions, it is probable that only half of it was blood. This bleeding was also arrested in a few minutes by pressure, and did not return.

For the purpose of knowing where to make the pressure, any light, flat, narrow, but firm substance should be prepared, such as a piece of cork, which can always be procured. The patient should then force all the coagulated blood out of the urethra; and as the bleeding usually takes place in these cases from that part which is anterior to the triangular ligament, pressure can readily be made upon it externally; but as it might be made a little before or behind the exact spot, in either of which cases it would be useless, the selection of the spot must be carefully attended to. This is done by beginning as far back as possible, and gradually bringing forward the finger by which the pressure is made. At a certain point the flow or dropping of blood will be arrested, and the precise spot from which it comes will be in all probability a little behind where the finger rests; a fact which can also be easily ascertained by carrying the finger a little backwards, when the blood will again flow. The bit of cork or pad can now be duly placed, and the patient should be desired to make pressure on it himself, which he can often more readily do than an assistant."

A chapter of the work is devoted to the consideration of impassable stricture, one of the most difficult and embarrassing cases in surgery.

The directions of our author for the management of this variety of stricture are replete with instruction, and if carried out with patience and discretion would have saved many a patient from the horrors of a painful and uncertain operation, and even from death itself. The rashness and haste often manifested, especially by the inexperienced Surgeon, to overcome an obstinate stricture, by severe and prompt treatment, has been the cause of so much mischief, and still prevails to so great extent, that we cannot avoid quoting Mr. Guthrie as authority for a milder and more certain course. On this point he thus expresses himself:

"When a stricture is impassable by the bougie, but is permeable by the urine, although it flows with difficulty, and there is no urgent necessity for the immediate removal of the obstruction, two different modes of proceeding may be adopted for its cure; one by a long continued and equable pressure, made on the face of the stricture by a pliable hollow gum elastic bougie, with which I have usually succeeded in overcoming the obstruction when not of any great extent; and of effecting a passage into the bladder, without giving rise to the alarm and anxiety which more vigorous measures sometimes occasion. The other, by steady pressure made for a short time at intervals with a solid instrument, until the obstruction is gradnally overcome.

It might be supposed that the continued presence of a bougie would give rise to a greater degree of irritation than previously existed, and in all probability to a complete retention of urine. It usually, however, calms the existing irritation, and after a few hours, if the patient becomes sensible of any difference, it is that his water passes more freely than before. The dilatation, nay, the mere separation of the sides of the urethra without any special dilatation, has an influence of a very favorable kind on a stricture, and may, without being carried further, effect a diminution of the contraction in slight cases, so as to allow a bougie to pass with little difficulty. In severe cases, the dilatation of the canal in front of a stricture does but little unless the dilating substance touches the stricture itself; a fact I have had proved, by finding that a bougie may remain for months in a false passage, beginning immediately in front of a stricture, without exerting upon it any perceptible influence.

The best dilating material is a pliable hollow gum elastic bougie, of a medium size, and perfectly smooth, and tolerably round at the point, so that it may give as little uneasiness as possible. This instrument is to be fixed in the urethra in the same way as a gum elastic catheter is fixed in the bladder; it should project about one inch beyond the orifice of the urethra, and rather less than more. The point should press against or rest upon the stricture with the greatest possible gentleness, so that it may not give rise to inflammation or ulceration, and yet should press just so much as to cause absorption. It is an admitted point in the animal economy, that new-formed parts, whether laid down in reparation or in disease, do not resist a stimulus in the same manner as parts of original formation. They are in fact removed by the action of the absorbents under the application of a stimulus, which has little or no influence on parts which have undergone no change, and are coeval with the existence of the individual. The pressure made by the point of the bougie should therefore be nicely regulated, so that it may do this and no more. The patient readily learns what is wanted, and as he can feel when the surgeon cannot, he soon understands how to manage the bougie himself, and can take it out, wash it, change it, or replace it, as he pleases. If he should be a very restless, fretful, or naturally irritable man, it may prevent sleep or prove inconvenient, in either of which cases it may be removed for two or three hours, at the pleasure of the individual, whose private affairs may otherwise render this indulgence necessary. If any further irritation should take place, it ought to be subdued by warm fomentations, by opiates, and perhaps by the application of a few leeches. There are few cases which require anything more, provided the patient will be perfectly quiet, live moderately, and preserve the recumbent position, until the irritation has subsided.

The principal and most satisfactory sign of amendment is the more ready flow of the urine; and although the bougie should not appear to advance, the improvement on this point is often progressive, until at last the bougie is either found to have passed through the stricture unknown to the patient, or is gently pressed through by his own hand, or by that of the surgeon. This object is effected in some cases in from three to six days; in others the progress is slow, although evident, and may require weeks; and in some, in which the obstruction is very hard and gristly, this method fails altogether, rendering the part more painful, by giving rise to inflammation in it, and to irritation in the bladder, requiring the removal of the instrument, and the abandonment of the practice. When it succeeds, and the canal is rendered pervious, the cure is only half completed, although the most difficult and dangerous part has been accomplished; the stricture has yielded in its centre, but not in its circumference. When the bougie has passed through the stricture, and the bladder is not in an irritable state, a catheter should be passed into it, as it is always a great satisfaction both to the patient and surgeon to see the urine flow through it."

In the large proportion of cases Mr. Guthrie has found this plan to succeed, and in speaking of the operation of dividing the perineum, he tells us—

"I have not had occasion to do this operation of late, and do not think I shall be obliged to resort to it; being under the belief that I have obtained so far the mastery of these obstructions as to be able to overcome them, in most instances, without having recourse to an incision in the perineum."

Chapters VI. and VII., "On suppression and retention of urine," and "On irritation of the membraneous and prostatic parts of the urethra" bear the same impress as those of which we have treated, but our limits admonish us to refrain from a further notice. Other important diseases of the urinary organs, not noticed in this volume, are contained in a second part of the same work.

